# Analyzing EEG of Quasi-Brain-Death Based on Dynamic Sample Entropy Measures

**DOI:** 10.1155/2013/618743

**Published:** 2013-12-22

**Authors:** Li Ni, Jianting Cao, Rubin Wang

**Affiliations:** ^1^East China University of Science and Technology, Meilong Road 130, Shanghai 200237, China; ^2^Saitama Institute of Technology, 1690 Fusaiji, Fukaya-shi, Saitama 369-0293, Japan; ^3^Brain Science Institute, RIKEN, 2-1 Hirosawa, Wako-shi, Saitama 351-0198, Japan

## Abstract

To give a more definite criterion using electroencephalograph (EEG) approach on brain death determination is vital for both reducing the risks and preventing medical misdiagnosis. This paper presents several novel adaptive computable entropy methods based on approximate entropy (ApEn) and sample entropy (SampEn) to monitor the varying symptoms of patients and to determine the brain death. The proposed method is a dynamic extension of the standard ApEn and SampEn by introducing a shifted time window. The main advantages of the developed dynamic approximate entropy (DApEn) and dynamic sample entropy (DSampEn) are for real-time computation and practical use. Results from the analysis of 35 patients (63 recordings) show that the proposed methods can illustrate effectiveness and well performance in evaluating the brain consciousness states.

## 1. Introduction

Brain death is defined as the complete, irreversible, and permanent loss of all brain and brainstem functions [1–4]. Under the definition, however, it is hard to conduct brain death judgement precisely for some clinical reasons. Traditional clinical tests are expensive, time consuming, and even dangerous in some cases (e.g., apnea test etc.). To avoid the above disadvantages, we have proposed an EEG preliminary examination procedure before the test of spontaneous respiration, which makes the test easier more effective and brings less risks [5]. To determine quasi-brain-death (QBD, where quasi means that it is a preliminary decision), EEG which is known to us as an important clinical tool for observing brain signals has been widely available in many countries to evaluate the absence of cerebral cortex function [5–7]. Our research aim is to provide several signal processing tools to determine brain death based on EEG analysis and help clinicians conduct the diagnosis in the practical operation.

The complexity of nonlinear physiologic signals has been wildly used in evaluating the differences between health and disease states [8]. The information of complexity contained by a physiologic time series directly reflects the state of such physiologic system [9]. The concept of entropy has been extensively available for complexity measures [10, 11]. Approximate entropy (ApEn) and sample entropy (SampEn) are effective approaches used in the complexity analysis and help us have a better understanding of biological system. Pincus first introduced ApEn [11], a set of measures of system complexity closely related to entropy, which has well been performed to analyze clinical cardiovascular and other time series. One defect of ApEn, however, is that its statistics lead to inconsistency. Therefore, Richman and Moorman have developed SampEn [12] as an improvement, due to that ApEn leads to bias where SampEn does not, which is caused by self matches, so that SampEn agrees with theory much more closely than ApEn over a broad range of conditions. In our studies, we will further illustrate the improved accuracy of SampEn statistics for brain death diagnosis.

This paper presents the dynamic extensions of ApEn and SampEn, since the static methods can only deal with a limited length of time series whereas the analysis of the data of long recording length is common in a biological system. The analysis on a small segment of the original data may probably cause a larger error and even a fault (e.g., the segment is seriously contaminated by noise), causing misleadingness. So that the dynamic method enables us to gain a more comprehensive and global view into a complex system. On the other hand, our dynamic method can decrease the amount of calculation in a simulation process and improve the efficiency for an analysis on a full data. As a result, the analysis on the successively changing information contained by a total time series is available for us.

The paper is organized as follows. In Section 2, we first recall a set of computable entropy methods including ApEn and SampEn and then derive the extension formulas of the proposed DApEn and DSampEn. In Section 3, we present real-world EEG data recording and data analysis results.  Section 4 includes the conclusions.

## 2. Methods of EEG Data Analysis

### 2.1. Approximate Entropy (ApEn)

In ApEn, the limited time series of *N* points, *U* = [*u*_1_, *u*_2_,…, *u*_*N*_] represented by the form of *m*-dimension vectors as *X*_*i*_ = [*u*_*i*_, *u*_*i*+1_,…, *u*_*i*+*m*−1_] and *X*_*j*_ = [*u*_*j*_, *u*_*j*+1_,…, *u*_*j*+*m*−1_], where *i*, *j* ≤ *N* − *m* + 1. The max distance between *X*_*i*_ and *X*_*j*_ can be calculated by

(1) $$\begin{array}{c} d\left[X_{i},X_{j}\right]=\underset{k=1,2,\ldots ,m}{\max}\left[\left|u_{i+k-1}-u_{j+k-1}\right|\right]. \end{array}$$

Given a threshold *r* and each *i* ≤ *N* − *m* + 1, let *B*_*i*_^*m*^ be the number of vectors *X*_*j*_ within *r* of *X*_*i*_, and we define

(2) $$\begin{array}{c} C_{i}^{m}\left(r\right)=\frac{B_{i}^{m}}{N-m+1},\;\text{where }i\leq N-m+1, \end{array}$$

and *ϕ* ^*m*^(*r*) as a mean of *C*_*i*_^*m*^(*r*)

(3) $$\begin{array}{c} \phi^{m}\left(r\right)=\frac{1}{N-m+1}\sum_{i=1}^{N-m+1}\ln C_{i}^{m}\left(r\right). \end{array}$$

Equation (2) is mainly defined to calculate the possibility that, for each *X*_*i*_ and *X*_*j*_, the two vectors are similar within the threshold *r*, while (3) is used to calculate the average.

By finding *ϕ* ^*m*+1^(*r*), ApEn(*r*, *m*, *N*) takes the form as

(4) $$\begin{array}{c} \text{ApEn}\left(m,r,N\right)=\phi^{m}\left(r\right)-\phi^{m+1}\left(r\right). \end{array}$$

This is how ApEn is defined to measure the self-similarity of the time series [11].

### 2.2. Sample Entropy (SampEn)

SampEn deals with the same *m*-dimension vectors *X*_*i*_ and *X*_*j*_ as defined in ApEn. The distance between two vectors is calculated by (1). In SampEn, let *A*_*i*_^*m*^ denote the number of vectors *X*_*j*_ within *r* of *X*_*i*_ times (*N* − *m*)^−1^, for *j* ranges from 1 to *N* − *m* + 1 and *j* ≠ *i*, excluding self-matches. We then define *A* ^*m*^ as a mean of *A*_*i*_^*m*^, for all 1 ≤ *i* ≤ *N* − *m* + 1, and takes the form as

(5) $$\begin{array}{c} A^{m}=\sum_{i=1}^{N-m+1}\frac{A_{i}^{m}}{N-m+1}. \end{array}$$

By increasing the space dimension to *m* + 1 and also repeating the steps in (1), (5), we can obtain *A* ^*m*+1^. Then SampEn can be obtained by

(6) $$\begin{array}{c} \text{SampEn}\left(m,r,N\right)=-\ln\left(\frac{A^{m+1}\left(r\right)}{A^{m}\left(r\right)}\right). \end{array}$$

This is how SampEn is defined to measure the self-similarity of the time series [11, 12].

### 2.3. Dynamic Extensions of ApEn and SampEn

**Figure 1:**
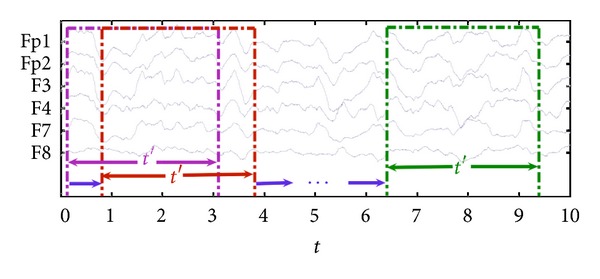
The diagram of dynamic measures. The time sections of 6 channels (Fp1, Fp2, F3, F4, F7, and F8) were intercepted as an example. Our dynamic method dealt with data in a moving time window with length *t*′.

The dynamic ApEn (DApEn) and dynamic SampEn (DSampEn) are proposed for the analyzing physiologic time series. The values of ApEn or SampEn are calculated in a set of consecutive time windows marked with their starting moments *t* with a length *t*′ of the whole data with a length *T*, respectively, as shown in Figure 1. Here, the expressions of DApEn and DSampEn are then obtained as ApEn(*m*, *r*, *N*)_*t*_ and SampEn(*m*, *r*, *N*)_*t*_, where the footnotes *t* represent the time windows for ApEn and SampEn computation. As a result, if the denoted variable *t* ranges, for example, from *t*_1_ to *t*_2_ with a step length *λ* (set *λ* = *t*′), the values of ApEn or SampEn are obtained, respectively, in several nonoverlapping windows. Then DApEn is defined by

(7) $$\begin{array}{c} \text{ApEn}{\left(m,r,N\right)}_{t}=\phi_{t}^{m}\left(r\right)-\phi_{t}^{m+1}\left(r\right), \end{array}$$

while DSampEn is defined by

(8) $$\begin{array}{c} \text{SampEn}{\left(m,r,N\right)}_{t}=-\ln\left(\frac{A_{t}^{m+1}\left(r\right)}{A_{t}^{m}\left(r\right)}\right). \end{array}$$

## 3. Experimental Results

In our present study, the EEG experimental protocols were executed in the ICUs of a hospital. The EEG data were directly recorded at the bedside of patient where high environmental noise from medical machines seriously corrupted recording procedure. The EEG recording instrument was a portable NEUROSCAN ESI-32 amplifier associated with a laptop computer. During EEG recording, a total of nine electrodes were placed on the forehead of the patients. Six channels were placed at corresponding electrodes (Fp1, Fp2, F3, F4, F7, and F8), two electrodes (A1, A2) were placed on the ears as reference and an additional channel, GND, served as the ground, whose sampling rates were all set as 1000 Hz and resistances of electrodes were set under 8 kΩ. Experimental data were obtained from 35 patients (19 male, 16 female) of ages ranging from 17 to 85 years old; 19 of them were diagnosed to be comatose and the left were brain deaths. The average length of the EEG recordings from these patients was about five minutes. Instead of using the EEG cap in the standard EEG recording, we used individual electrodes with high-chloride electrolyte gel (suitable for using DC power) in all data recording sessions.

### 3.1. Comparison of ApEn and SampEn

The EEG signals of the coma cases include brain waves together with environmental noise, while the EEG signals of quasi-brain-death only include environmental noise. Therefore, we consider that regular or predictable components exist in the EEG signals of coma patients. Both ApEn and SampEn measures of coma states may assign a much lower value to coma cases.

Let us begin with having a look into the tables, where ApEn and SampEn results of two patients, respectively, in the state of coma (C) and in the state of brain death (D) for each channel with different threshold are given. In general, we took samples of 100–5000 points, set *m* = 2, and the tolerance as *r* = 0.15 × standard deviation (SD) or *r* = 0.25 × SD according to [11, 12]. In order to demonstrate the differences between the two types, we present one case from each group.

From Tables 1 and 2, we find that the calculated values of both ApEn and SampEn significantly differ between the coma and the brain deaths. On the other hand, *d* denoted as the average difference indicates the capability to classify the two states. Particularly in the SampEn case with the threshold *r* = 0.15 × SD, there is a relatively huger difference between these two states. In a word, here we regard SampEn as a method in the first place. To eliminate the possibility that the divergence is caused by different patients, we take another special example for a certain patient as a further result in the Table 3. This patient behaved from coma to brain death.

Obviously, the results of both ApEn and SampEn for this certain patient who behaved these two states are tremendously different, which means that it is an available and significant method for us to carry on brain death diagnosis.

### 3.2. Statistical Results

**Figure 2:**
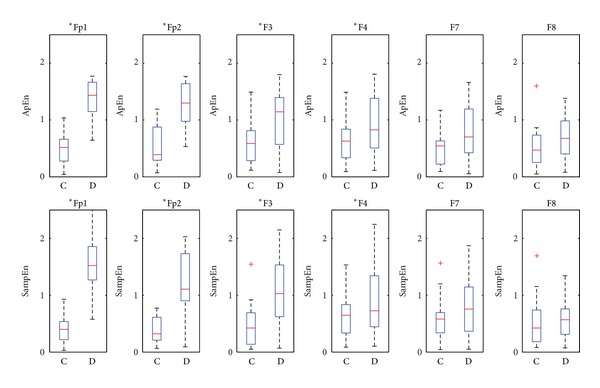
Statistical results for coma state (C) and brain death state (D).

To obtain more convincing results, ApEn and SampEn are applied to calculate all our recordings. As shown in Figure 2, the box plot of ApEn and SampEn are calculated for all the six channels with the same parameters. According to the two-way ANOVA against the null hypothesis that the two groups have the same mean and the returned *p* value, the significance is found and the null hypothesis is rejected (*H* = 1) for our EEG experiment. Note that the performance of each electrodes is different because of the different distance to the reference, and comparison of complexity measures across each channel is also given by two-way ANOVA that summarized in Table 4. As seen, the results obtained from channel Fp1 and Fp2 are more reliable and convincible for our brain death diagnosis according to the box plot. For the other channels, the determination of coma and brain death states is statistically clear; however, the values of both states range within a common section, possibly causing misdiagnosis for one patient. So our further effort is based on the data sampled through channel Fp1 or Fp2. *t*-test is also used to show the significance (*P* < 0.05) for the two groups with the marker  *  for each channel in Figure 2.

### 3.3. Results for DApEn and DSampEn

**Figure 3:**
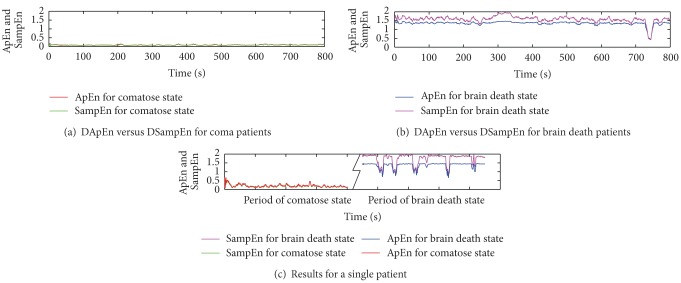
Dynamic complexity measure for two different cases (a) versus (b) and a patient has two states (c).

For the real-time application such as monitoring a state of the patient, it is necessary to introduce dynamic-based analysis to explore the brain wave activity changes of the patients over time. As shown in Figure 3, over the time coordinate (0–800 s) of EEG signals, ApEn and SampEn in each second are calculated. Results of DApEn and DSampEn for a coma case (a), a quasi-brain-death case (b), and a case that the patient behaved from coma to quasi-brain-death (c).

**Figure 4:**
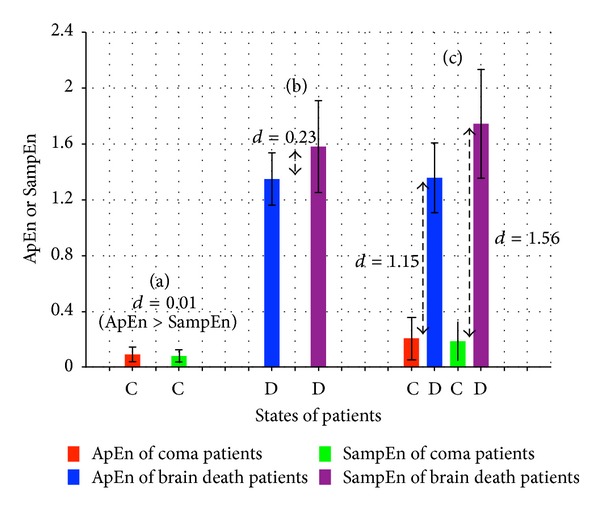
Based on the obtained results in Figure 3, the corresponding average values and error bars are calculated and summarized as supplementary information.

Under the same experimental environment, the results of DApEn and DSampEn are obtained by (7) and (8). To obtain a more smooth curve, the moving average method is applied to decrease the high frequency part by calculating the average of every ten points. For the same coma case, values of SampEn (green) remain low over time, while values of ApEn (red) remain slightly higher than those of SampEn. For the same quasi-brain-death case, SampEn assigns a higher value (purple) than that of ApEn (blue). This indicates a more powerful capability for DSampEn to classify the two brain consciousness states than for ApEn. Around 750 s, the huge fluctuation is caused by the serious contamination of noise. Moreover, the results of DApEn and DSampEn of the certain patient whose coma state and brain death state are both recorded are plotted in Figure 3(c). In this case, this patient's two states are well discernible because of a huge difference of the values of both DApEn and DSampEn, however, the results of coma state are slightly different and several data segments are under the influence of interfering noise. To make our estimates in Figure 3 solid, we would like to give the corresponding average values and error bars in Figure 4. We define *d* as the average distance between the bars which estimates the difference between the two methods and also the capability of states determination. It also helps prove that DSampEn value of coma state and its value of brain death state vary more greatly than DApEn values which means DSampEn is better to discern the states of coma and brain death. As an expansion of the standard SampEn, DSampEn is more durably effective to perform a function.

From all the obtained results, we firmly believe that complexity of the coma and the brain deaths can be well used for brain consciousness determination. The plotted dynamic ApEn or SampEn indicates the state of a patient and such time-dependent methods also help monitor the trend of a patient's state, with which clinic can carry out emergency medical care before danger. But in this paper the methods applied to predict are beyond our scope. So, with the help of our dynamic algorithm, the online EEG preliminary brain death determination system comes into reality.

## 4. Conclusions

This paper has proposed the recently introduced complexity analysis based on calculating entropy of a time series in physiologic systems. For approximate entropy and sample entropy measures, we have found a complexity-analysis-based criterion on the determination of quasi-brain-death, providing a reference for our proposed EEG preliminary procedure. Furthermore, based on the criterion, we have tested all our recordings and obtain the statistically reliable result which is identical to the clinical brain death determination completely. Finally, we have developed the novel DApEn and DSampEn algorithms for the online EEG preliminary brain death determination system. These methods may be applied to analysis of other physiologic time series as a reference.

## Figures and Tables

**Table 1:** Set *r* = 0.15 × SD, with this threshold, and calculate the average difference *d* for each channel. In the ApEn case, *d* = 0.69, while in the SampEn case, *d* = 0.97.

ApEn case	Fp1	Fp2	F3	F4	F7	F8	Avg.	Std.
C	0.5502	0.4361	0.3974	0.2999	0.9083	0.4221	0.5023	0.2144
D	1.0656	1.0624	1.2173	1.4059	1.3344	1.0644	1.1917	0.1521

SampEn case	Fp1	Fp2	F3	F4	F7	F8	Avg.	Std.

C	0.5005	0.3618	0.3195	0.2403	0.9054	0.3204	0.4413	0.2429
D	1.1806	1.2222	1.5216	1.7924	1.5891	1.1719	1.4130	0.2590

**Table 2:** Set *r* = 0.25 × SD, with this threshold, and calculate the average difference *d* for each channel. In the ApEn case, *d* = 0.71, while in the SampEn case, *d* = 0.75.

ApEn case	Fp1	Fp2	F3	F4	F7	F8	Avg.	Std.
C	0.3215	0.2336	0.1977	0.1415	0.6265	0.2279	0.2914	0.1743
D	0.8232	0.8353	1.0132	1.3042	1.1760	0.8363	0.9980	0.2044

SampEn case	Fp1	Fp2	F3	F4	F7	F8	Avg.	Std.

C	0.2802	0.1926	0.1588	0.1178	0.6146	0.1655	0.2549	0.1844
D	0.8036	0.8122	1.0239	1.3749	1.2068	0.8182	1.0066	0.2410

**Table 3:** Approximate entropy and sample entropy results of a certain patient in the state of coma (C) and brain death (D) for each channel with threshold *r* = 0.15 × SD, *d*_apen_ = 1.11, *d*_sampen_ = 1.20.

ApEn case	Fp1	Fp2	F3	F4	F7	F8	Avg.	Std.
C	0.1637	0.3323	0.8113	0.4469	0.4955	0.0989	0.3914	0.2573
D	1.7701	1.7669	1.6229	1.3967	1.2260	1.2009	1.4973	0.2586

SampEn case	Fp1	Fp2	F3	F4	F7	F8	Avg.	Std.

C	0.1523	0.3009	0.7261	0.3972	0.4480	0.0914	0.3527	0.2287
D	1.9952	2.0280	1.6697	1.3353	1.1699	1.1090	1.5512	0.4065

**Table 4:** The two-way ANOVA results for two groups and for the performance across channels.

ANOVA	Coma versus brain death	Channel
ApEn cases	*p* = 1.3 × 10^−53^, *H* = 1	*p* = 2.3 × 10^−6^, *H* = 1
SampEn cases	*p* = 2.5 × 10^−52^, *H* = 1	*p* = 1.8 × 10^−8^, *H* = 1
